# Sexual Orientation and Cost-Related Health Care Deferral

**DOI:** 10.1001/jamanetworkopen.2025.49101

**Published:** 2025-12-23

**Authors:** Alexandra Balshi, John P. Dempsey, Hannah R. Thompson, Mary W. Montgomery

**Affiliations:** 1 Harvard Medical School, Boston, Massachusetts; 2 Norton College of Medicine, State University of New York Upstate Medical University, Syracuse; 3 Department of Community Health Sciences, School of Public Health, University of California, Berkeley, Berkeley; 4 Department of Infectious Diseases, Brigham and Women’s Hospital, Boston, Massachusetts

## Abstract

This survey study assesses associations between lesbian, gay, bisexual, or other sexual minority identity and difficulty affording medical care or deferral of health care among US adults.

## Introduction

Survey data from 2014 to 2017 revealed that, compared with heterosexual individuals, lesbian, gay, bisexual, and other nonheterosexual (LGB+) adults are more likely to forgo necessary medical care due to cost, even when insured.^1,2^ Since then, the US political, legislative, and sociocultural milieu has shifted^3^ such that LGB+ cost-related health care deferral must be reevaluated. For example, the Trump administration attempted to limit the Affordable Care Act Section 1557 nondiscrimination protections, which the Biden administration later worked to enshrine.^4^ Extensive research documents economic disparities facing sexual minority households, which contribute to disproportionate difficulties in affording health care.^5,6^ Given recent rollbacks of LGB+ legal protections, we hypothesize an increase in such difficulty. In this study, we used a nationally representative sample of US adults to explore associations between LGB+ identity, difficulty affording medical care, and care deferral.

## Methods

We classified 2019 to 2023 National Health Interview Survey (NHIS) participants (aged ≥18 years) as LGB+ or heterosexual based on sexual-orientation question responses. In accordance with the Common Rule (45 CFR § 46.104), this survey study was exempt from ethics review and consent requirement because deidentified, public data were used. We followed the AAPOR reporting guideline.

Participants self-reported whether, in the past year, they needed but could not afford medical care, prescription medications, and mental health care and whether they delayed filling prescriptions or skipped medication doses to save money. Logistic regression models ascertained associations between LGB+ identity and these 5 study outcomes using 4 iterative models: unadjusted; adjusted for social determinants of health (SDOH: age, sex, race and ethnicity, education, employment, marital status, income, citizenship status, health care access, residency); adjusted for SDOH plus self-reported health status and disability; and adjusted for SDOH, health status, and insurance coverage and type. Subanalysis (model 4a) included model 4 adjustments plus high-deductible health plan (HDHP) enrollment. To account for multiple comparisons, we used Bonferroni-corrected significance of *P* = .01.

All analyses used survey weights with the svy command in Stata, v18.0 (StataCorp). The eMethods and eFigure in Supplement 1 provide additional details.

## Results

We included 132 013 participants (mean [SD] age, 47.3 [16.4] years; 67 694 females [51.3%]), representing 238 665 755 adults (Table 1). Overall, 127 033 participants (96.2%) identified as heterosexual, 2605 (2.0%) as lesbian or gay, and 2423 (1.8%) as bisexual.

**Table 1.** Population Characteristics of Participants in the 2019 to 2023 National Health Interview Survey by Sexual Orientation

Variable	Participants, No. (%)		*P* value^a^
	LGB+	Heterosexual	
No. of participants	5028	127 033	NA
Age group, y			
18-24	1398 (27.8)	13 681 (10.8)	<.001
25-34	1506 (30.0)	21 443 (16.9)	<.001
35-44	794 (15.8)	21 049 (16.6)	.21
45-54	531 (10.6)	20 389 (16.1)	<.001
55-64	458 (9.1)	21 621 (17.0)	<.001
≥65	340 (6.8)	28 849 (22.7)	<.001
Sex			
Female	3022 (60.2)	64 672 (50.9)	<.001
Male	2006 (39.8)	62 361 (49.1)	<.001
Race^b^			
American Indian or Alaska Native	61 (1.2)	825 (0.7)	.001
Asian	152 (3.0)	7711 (6.1)	<.001
Black or African American	542 (10.8)	14 596 (11.5)	.27
White	3343 (66.5)	80 043 (63.0)	<.001
Other^c^	143 (2.8)	1448 (1.1)	<.001
Ethnicity^b^			
Hispanic or Latino	746 (14.8)	21 532 (17.0)	.003
Non-Hispanic or Latino	4282 (85.2)	105 501 (83.1)	.003
Highest educational level			
<College degree	3242 (64.3)	86 368 (67.9)	<.001
≥College degree	1786 (35.7)	40 665 (32.2)	<.001
Employment status			
Employed	3566 (71.0)	79 550 (62.8)	<.001
Household income to poverty threshold ratio^d^			
<1	633 (12.6)	12 462 (9.8)	<.001
1 to <2	869 (17.4)	22 739 (17.9)	.47
≥2	3519 (70.0)	91 819 (72.3)	.005
Health care access			
Has a usual place	4,33 (86.2)	114 122 (89.9)	<.001
Place of residence			
Metropolitan	4558 (90.7)	108 931 (85.8)	<.001
Nonmetropolitan	470 (9.4)	18 102 (14.3)	<.001
Married or partnered	2227 (44.4)	78 293 (61.8)	<.001
US citizenship	4879 (97.2)	116 093 (91.7)	<.001
Health insurance			
Covered	4499 (89.7)	114 015 (90.0)	.71
Health insurance type			
Uninsured	516 (10.3)	12 739 (10.1)	.71
Private	3232 (64.5)	81 039 (64.0)	.61
Military	181 (3.6)	6701 (5.3)	.002
Medicaid	932 (18.6)	15 851 (12.5)	<.001
Medicare	488 (9.7)	31 087 (24.5)	<.001
Children’s program	0	0	.70
Other state-sponsored	31 (0.6)	747 (0.6)	.71
Other government	5 (0.1)	304 (0.2)	.045

Abbreviations: LGB+, lesbian, gay, bisexual, and other nonheterosexual orientation; NA, not applicable.

^a^ *P* values determined from unadjusted linear and logistic regression models with National Health Interview Survey sampling weights.

^b^ Race and ethnicity data were self-reported and included because of well-established racial and ethnic disparities in health care access in the US.

^c^ Other race included Native Hawaiian or Pacific Islander and self-reported “some other race.”

^d^ The National Health Interview Survey compares participant-reported family income with the federal poverty threshold. We used the family income to poverty threshold ratio in our analyses, as recommended by the US Census Bureau.

All models demonstrated LGB+ individuals faced greater difficulty in affording necessary medical care (Table 2). Under model 4, LGB+ participants had higher odds than heterosexual individuals of needing but being unable to afford medical care (odds ratio [OR], 1.76; 95% CI, 1.52-2.02), prescription medications (OR, 1.71; 95% CI, 1.50-1.95), and mental health care (OR, 2.73; 95% CI, 2.43-3.07) as well as delaying prescription fills (OR, 1.46; 95% CI, 1.27-1.69) and skipping doses (OR, 1.45; 95% CI, 1.23-1.70) to save money. Our subanalysis including HDHPs replicated these findings, except for skipping doses.

**Table 2.** Adjusted Odds Ratio of Difficulty Affording Medical Care for LGB+ and Heterosexual Participants in the 2019 to 2023 National Health Interview Survey^a^

Study outcome	Participants, No. (%)		OR (95% CI)				
	LGB+	Heterosexual	Model 1 (Unadjusted)	Model 2 (Adjusted for SDOH)	Model 3 (Adjusted for SDOH and health status)	Model 4 (Adjusted for SDOH, health status, and insurance)	Model 4a (Model 4 adjustments + HDHP)
No. of participants	5028	127 033	132 013	130 609	130 563	130 311	51 534
Could not afford necessary medical care	559 (11.1)	7508 (5.9)	2.15 (1.90-2.43)	1.91 (1.68-2.17)	1.63 (1.43-1.87)	1.76 (1.52-2.02)	1.71 (1.37-2.14)
Could not afford necessary prescription medications	520 (10.3)	6767 (5.3)	2.16 (1.92-2.43)	1.98 (1.75-2.25)	1.66 (1.46-1.90)	1.71 (1.50-1.95)	1.72 (1.41-2.09)
Could not afford necessary mental health care	770 (15.3)	4911 (3.9)	4.98 (4.49-5.53)	3.08 (2.75-3.45)	2.68 (2.38-3.01)	2.73 (2.43-3.07)	2.67 (2.25-3.18)
Delayed filling prescriptions due to cost	428 (11.5)	5112 (5.7)	2.08 (1.82-2.38)	1.64 (1.42-1.89)	1.45 (1.25-1.67)	1.46 (1.27-1.69)	1.43 (1.16-1.77)^b^
Skipped medication doses due to cost	297 (8.0)	3640 (4.0)	2.06 (1.77-2.39)	1.62 (1.38-1.91)	1.43 (1.22-1.68)	1.45 (1.23-1.70)	1.33 (1.04-1.70)^c^

Abbreviations: HDHP, high-deductible health plan; LGB+, lesbian, gay, bisexual, and other nonheterosexual orientation; OR, odds ratio; SDOH, social determinants of health.

^a^ ORs were determined from logistic regression models with National Health Interview Survey sampling weights.

^b^ *P* = .001.

^c^ *P* = .03.

## Discussion

After accounting for income and other SDOH and insurance variables, LGB+ participants had disproportionally higher odds across all outcomes, deferring necessary medical care due to cost. These odds were nearly 3-fold greater than for heterosexual individuals, a sobering finding given the increased burden of mental illness in the LGB+ community.

Study limitations include use of self-reported surveys, which are susceptible to recall and response biases; inability to perform subanalyses by individual sexual-minority identity due to sample size limitations; inability to assess outcome differences among transgender and gender-diverse populations; and potential unobserved confounding due to lack of robust measures of clinical burden, insurance, and state policy.

The medical community can bridge these gaps by inquiring about financial barriers during clinical encounters and proactively connecting patients to resource support to mitigate costs. Centers for Medicare & Medicaid Services can restore continuous eligibility, reduce churn, and strengthen grievance processes. Legislators must protect Section 1557 and enact policies that cap out-of-pocket spending. These efforts are more urgent than ever before given the current American sociopolitical climate and escalating attacks on LGB+ rights.

## Notes

Supplement 1.
eMethods
**eFigure.** Flow Diagram of Participant Inclusion and Exclusion
eReferences

Supplement 2.
Data Sharing Statement
