# Supplementary material for: Sexual Orientation and Cost-Related Health Care Deferral
Source: JAMA Netw Open. 2025 Dec 23;8(12):e2549101. doi: 10.1001/jamanetworkopen.2025.49101 (PMC12728644; doi:10.1001/jamanetworkopen.2025.49101)
Supplement: Supplement 1. — eMethods eFigure. Flow Diagram of Participant Inclusion and Exclusion eReferences [file jamanetwopen-e2549101-s001.pdf]

Supplemental Online Content

Balshi A, Dempsey JP, Thompson HR, Montgomery MW. Sexual orientation and cost-related health care deferral. *JAMA Netw Open*. 2025;8(12):e2549101. doi:10.1001/jamanetworkopen.2025.49101

eMethods

eFigure. Flow Diagram of Participant Inclusion and Exclusion

eReferences

This supplemental material has been provided by the authors to give readers additional information about their work.

eMethods

Data Source and Cohort Classification

Pooled, cross-sectional National Health Interview Survey (NHIS) data from 2019 to 2023 were extracted from the Integrated Public Use Microdata Series website. The NHIS employs a complex multistage probability design to ensure nationally representative estimates, accounting for nonresponse and oversampling underrepresented groups. Data were drawn from the Sample Adult files, which include detailed health information collected through in-depth interviews with one randomly selected adult per household, aged 18 years or older.

Participants were asked which of the following best represented how they thought of themselves: (1) lesbian or gay; (2) straight, that is, not lesbian or gay; (3) bisexual; (4) something else; (5) I don't know the answer; or (6) refuse. We excluded those who "did not know the answer", those who answered "something else," and those who refused. We then classified the cohort as LGB (lesbian or gay or bisexual) or heterosexual.

Study Covariates

Covariates—age, sex, race, ethnicity, education, employment, marital status, income[1], citizenship, health status, healthcare access, residential setting, disability status (Washington Group Short Set on Functioning[2]), and insurance information—were chosen a priori given documented links to socioeconomic and cost-related care deferral.[3-10]

Sex, Race, and Ethnicity

Participants self-reported sex by answering the question "Are you male or female?" and self-reported race by selecting "one or more of these categories: White, Black, African American, American Indian, Alaska Native, Native Hawaiian, Pacific Islander, Asian, or some other race?". Ethnicity was also self-reported with the instructions: "Do you consider yourself to be Hispanic or Latino?".

Education, Employment, and Income

Participants responded to the following question to report their highest level of educational attainment: "What is the HIGHEST level of school you have completed or the highest degree you have received?". We divided our sample into two groups, those who had received a bachelor's degree or greater and those who had not. To classify employment status, participants reported employment status in past 1 to 2 weeks (employed or not). Participants also report family income which the NHIS then compares to the appropriate federal poverty threshold. We used the ratio of ratio of family income to the poverty threshold in our analyses.

Healthcare Access, Insurance Coverage, Health Status, and Place of Residence

To determine access to healthcare, participants were asked "Is there a place that you USUALLY go to if you are sick and need health care?" which we classified as yes or no. Health insurance coverage was defined as covered or not covered. Participants self-reported their health status on a 5-point scale ranging from excellent to poor. Place of residence was classified by county as large central metro, large fringe metro, medium and small metro, and nonmetropolitan.

Marital Status

Marital status was defined by answers to the question "Are you now married, living with a partner together as an unmarried couple, or neither?". We equated being married or living with a partner in our analyses.

US Citizenship

US Citizenship was asked in a yes, no manner, asking are you "a citizen of the United States?".

© 2025 Balshi A et al. *JAMA Network Open*

Insurance

Insurance type was classified as Uninsured, Private, Military, Medicaid, Medicare, Children's Program, Other State-Sponsored, or Other Government. Participants with private health insurance were asked "A deductible is the amount you have to pay for health care before your health insurance or health coverage plan will start paying your medical bills. Does [your] health plan have an annual deductible?". For those who answered yes, the threshold for a high deductible was $1,500 for a plan that covers only one person and $3,000 for a plan that covers two or more persons.

Statistical analysis

All analyses utilized survey weights with the svy command in Stata (Stata v18.0, StataCorp LLC) to account for the NHIS's complex sampling design and ensure nationally representative estimates. Following guidance from the National Center for Health Statistics (NCHS), weights were pooled and divided by the number of years studied. Descriptive statistics were used to summarize and characterize the study sample. Records with missing values on any variable of interest were excluded from our analyses (**eFigure**).

eFigure. Flow Diagram of Participant Inclusion and Exclusion

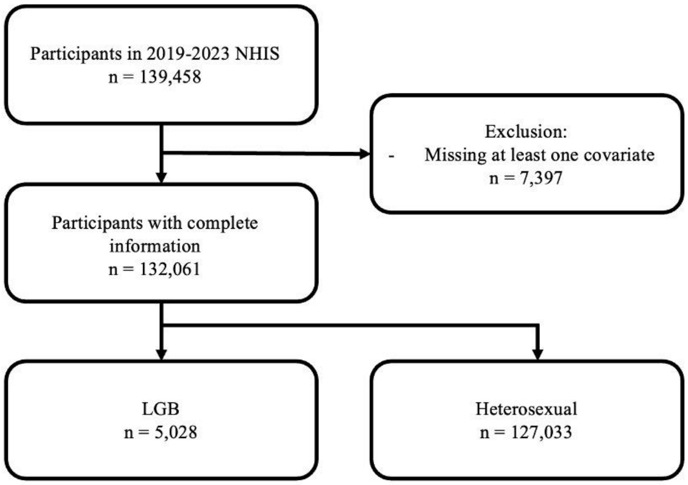

Abbreviations: NHIS, National Health Interview Survey; LGB, lesbian or gay or bisexual.

Flow diagram of sample selection from the National Health Interview Survey (2019-2023) illustrating how the final analytic cohort for cost-related care deferral was arrived at.

Study limitations

This study has several important limitations. First, all study outcomes were assessed through self-reported surveys and thus were susceptible to both recall and response biases. Second, we were unable to perform subanalyses of individual sexual minority identities (lesbian, gay, or bisexual) due to sample size limitations, which may obscure differences in healthcare access and affordability within the LGB population. Third, this study could not evaluate outcome differences among transgender and gender-diverse populations, as this data is restricted by the NCHS. Fourth, unobserved confounding is possible as we lacked robust measures of clinical burden (multimorbidity beyond self-reported health and disability), insurance generosity (deductible amount, copays, out-of-pocket max, network breadth), and state policy environment (e.g., Medicaid expansion, LGB protections), the latter unavailable in NHIS public-use files.

© 2025 Balshi A et al. *JAMA Network Open*

eReferences

1. US Census Bureau. How the Census Bureau Measures Poverty. Census.gov. Accessed September 15, 2025. https://www.census.gov/topics/income-poverty/poverty/guidance/poverty-measures.html
2. WG Short Set on Functioning (WG-SS) - The Washington Group on Disability Statistics. Accessed September 15, 2025. https://www.washingtongroup-disability.com/question-sets/wg-short-set-on-functioning-wg-ss/
3. Mahajan S, Caraballo C, Lu Y, et al. Trends in Differences in Health Status and Health Care Access and Affordability by Race and Ethnicity in the United States, 1999-2018. *JAMA*. 2021;326(7):637–648. doi:10.1001/jama.2021.9907
4. Scherer AM, Solway E, Malani PN, et al. Factors Associated with Health Insurance Affordability Concerns Among U.S. Adults Age 50-64: a Cross-Sectional, Nationally Representative Study. *J Gen Intern Med*. 2021;36(2):546-548. doi:10.1007/s11606-020-05732-2
5. Tipirneni R, Roberts ET, Levy HG, et al. Health Care Utilization and Costs for Older Adults Aging Into Medicare After the Affordable Care Act. *JAMA Health Forum*. 2025;6(1):e245025. Published 2025 Jan 3. doi:10.1001/jamahealthforum.2024.5025
6. Park S, Stimpson JP. Health Care Expenses and Financial Hardship Among Medicare Beneficiaries With Functional Disability. *JAMA Netw Open*. 2024;7(6):e2417300. Published 2024 Jun 3. doi:10.1001/jamanetworkopen.2024.17300
7. Okoro UE, Harland KK, Assimacopoulos E, Findlay S. Trends in Health Care Coverage and Out-Of-Pocket Cost Barriers: A Gender Comparison. *J Womens Health (Larchmt)*. 2024;33(4):473-479. doi:10.1089/jwh.2023.0121
8. Simpson JL, Cohen RA. The Association of Marital Status and Offers of Employer-based Health Insurance for Employed Women Aged 27-64: United States, 2014-2015. *NCHS Data Brief*. 2017;(268):1-8.
9. MacDougall H, Mork D, Hanson S, Smith CH. Rural-urban differences in health care unaffordability. *J Rural Health*. 2024;40(2):376-385. doi:10.1111/jrh.12788
10. Bolibol A, Buchmueller TC, Lewis B, Miller S. Health insurance coverage and access to care among LGBT adults, 2013-19. *Health Aff.* 2023;42(6):858-65.

© 2025 Balshi A et al. *JAMA Network Open*
