# Supplementary material for: Sexual Orientation and Cost-Related Health Care Deferral
Source: JAMA Netw Open. 2025 Dec 23;8(12):e2549101. doi: 10.1001/jamanetworkopen.2025.49101 (PMC12728644; doi:10.1001/jamanetworkopen.2025.49101)
Supplement: Supplement 2. — Data Sharing Statement [file jamanetwopen-e2549101-s002.pdf]

Data Sharing Statement

Balshi. Sexual Orientation and Cost-Related Health Care Deferral. *JAMA Netw Open*.
Published December 23, 2025. doi:10.1001/jamanetworkopen.2025.49101

Data
Data available: No

Additional Information
Explanation for why data not available: Data is publicly available
